# Severe and moderate seasonal influenza epidemics among Italian healthcare workers: A comparison of the excess of absenteeism

**DOI:** 10.1111/irv.12777

**Published:** 2020-07-14

**Authors:** Maria Michela Gianino, Omar Kakaa, Gianfranco Politano, Antonio Scarmozzino, Alfredo Benso, Carla Maria Zotti

**Affiliations:** ^1^ Department of Sciences of Public Health and Pediatrics University of Torino Torino Italy; ^2^ Department of Control and Computer Engineering Politecnico di Torino Torino Italy; ^3^ AOU Città della salute e della Scienza Torino, Torino Italy

**Keywords:** absenteeism, healthcare workers, moderate seasonal influenza, seasonal influenza epidemics, severe seasonal influenza, vaccination

## Abstract

**Background:**

This study aims to quantify the excess of sickness absenteeism among healthcare workers (HCWs), to estimate the impact of a severe versus moderate influenza season and to determine whether the vaccination rates are associated with reduced sickness absence.

**Methods:**

We investigated the excess absenteeism that occurred in a large Italian hospital, 5300 HCWs, during the severe influenza season of 2017/2018 and compared it with three moderate flu seasons (2010/2013). Data on influenza vaccinations and absenteeism were obtained from the hospital's databases. The data were split into two periods: the epidemic, from 42 to 17 weeks, and non‐epidemic, defined as 18 to 41 weeks, which was used as the baseline. We stratified the absenteeism among HCWs in multiple variables.

**Results:**

Our study showed an increased absenteeism among HCWs during the epidemic period of severe season in comparison with non‐epidemic periods, the absolute increase correlated with a relative increase of 70% (from 4.05 to 6.68 days/person). Vaccinated HCWs had less excess of absenteeism in comparison with non‐vaccinated HCWs (1.74 vs 2.71 days/person). The comparison with the moderate seasons showed a stronger impact on HCW sick absenteeism in the severe season (+0.747days/person, *P* = .03), especially among nurses and HCWs in contact with patients (+1.53 *P* < .01; +1.19 *P* < .01).

**Conclusions:**

In conclusion, a severe influenza epidemic has greater impacts on the absenteeism among HCWs than a moderate one. Although at a low rate, a positive effect of vaccination on absenteeism is present, it may support healthcare facilities to recommend vaccinations for their workers.

## 1 INTRODUCTION

In March 2019, the WHO published the “*The Global Influenza Strategy for 2019‐2030,”* with the goal of strengthening seasonal prevention and control and preparedness for future pandemics. Seasonal influenza viruses continuously evolve and cause severe disease annually, particularly in older people, children, pregnant women and people with underlying chronic conditions. Each year, throughout the world, there are an estimated 1 billion cases of influenza, of which 3‐5 million are severe cases and 290 000‐650 000 lead to influenza‐related respiratory deaths. Outbreaks of influenza highlight the burden and severity of annual epidemics on the global population and on countries’ health systems.^1^

Consistent with the “*The Global Influenza Strategy for 2019‐2030”* goals 2B, 2C and 3B, our study attempts to assess the burden of influenza (flu) on absenteeism among healthcare workers (HCWs).^1^ HCWs are an essential element for the efficient delivery of quality health services to a community. Acute respiratory infections (ARIs) and influenza‐like illness (ILI) are the most common infectious causes of sickness absenteeism among workers^2^, ^3^ and can contribute to significant productivity loss and disruption of healthcare services during annual epidemics and occasional pandemics,^4^ periods characterized by an increased demand for healthcare assistance.^5^

Some studies provide evidence of increased flu absenteeism during periods of seasonal epidemics among HCWs,^6^, ^7^, ^8^ and after the 2009 influenza pandemic, some publications compared work absenteeism related to the pandemic with absenteeism during periods of seasonal epidemics.^9^ A study in Hong Kong found that influenza epidemics prior to the 2009 pandemic and during the 2009 pandemic were associated with 8.4% and 57.7% increases in overall sickness absence,^9^ respectively, and in a study in Norway,^10^ the absenteeism rates during the 2009 H1N1 pandemic period were estimated to have increased 1.5‐fold over the absenteeism rate during the 2005/2006 seasonal epidemic.

Because healthcare workers are at increased risk for acquisition of influenza, vaccination of HCWs can be justified by the need to protect them from occupational exposure to decrease related absenteeism and to protect their patients, who may not develop a satisfactory immune response after they are vaccinated (eg immunocompromised persons), may not be eligible for vaccination (eg influenza vaccines are not licensed for infants <6 months old) or may be unvaccinated because of missed opportunities or anti‐vaccination opinions.^11^

The Centers for Disease Control and Prevention of Atlanta (CDC) widely recommends annual flu vaccination of healthcare workers (HCWs) as the best way to prevent the disease and to avoid the transmission of influenza from staff to patients and from patients to staff.^12^

Annual influenza vaccinations are advocated for HCWs in many European countries, such as the UK, Germany, France and Spain, and HCWs in the medical field receive vaccinations more often than the general population, with percentages ranging from 15.6% to 63.2%.^13^ The rate of vaccination is even stronger in Canada, ranging from 50% to 69%^14^ and in Australia, up to 50%.^15^ The Italian Ministry of Health, in agreement with international guidelines, annually recommends vaccination for seasonal influenza to all healthcare workers (HCWs).^16^ Despite that, an Italian systematic review designed to estimate the pooled prevalence of influenza vaccinations among nurses and ancillary workers in Italy showed that the mean prevalence appears low compared with other European countries.^17^

Additional evidence indicating that influenza vaccination has a positive effect on healthcare workers’ own well‐being might further influence Italian healthcare workers’ beliefs and behaviours with respect to being vaccinated. We therefore aimed (i) to quantify the increase in absenteeism among HCWs at a large Italian hospital that occurred during severe intensity seasonal flu periods, (ii) to estimate the different impacts that a moderate intensity flu epidemic and a severe intensity epidemic can have on HCWs’ sick absenteeism and (iii) to examine the hypothesis that Italian health workers’ influenza vaccination rates are associated with reduced sickness absence.

## 2 MATERIALS AND METHODS

### 2.1 Design

In this study, we investigated the excess of absenteeism that occurred during the flu season of 2017‐2018, which is characterized as a severe intensity epidemic.^18^ The setting was the Azienda Ospedaliera Universitaria (AOU) “Città della salute e della Scienza,” a complex of four interconnected hospitals, and we focused on the HCWs of the Molinette Teaching Hospital, the largest of the four hospitals, which includes approximately 5300 workers (approximately 45% of the centre's employees).

To guarantee the comparability of the results and to estimate the different impacts of a moderate intensity flu epidemic and a severe intensity epidemic, the methodology was identical to a previous study^6^ in which we analysed data from the three consecutive years following the 2009 influenza pandemic, 2010‐2011, 2011‐2012 and 2012‐2013, during which seasonal influenza outbreaks were of moderate intensity.^19^ The qualification of severe and moderate intensity is based on the evaluation of the thresholds obtained with the MEM method.^20^ We will briefly explain the main key points.

All the data sets were obtained from hospital registers, national and regional reports, and they were subsequently merged and analysed.

The data for the Italian influenza epidemics were obtained from the national report of InfluNet (the Italian sentinel influenza surveillance network).^18^ The data from the report ranged from week 42 to week 17; in this period, sentinel physicians reported the weekly number of patients with ILI, ARI or both to the national centre for influenza surveillance. We also gathered ILI morbidity data from the regional epidemiological service (SEReMI) and compared them with the absenteeism rates, which allowed us to be more accurate from an epidemiological point of view.

Data on influenza vaccination for each employee were obtained from the Occupational Health Unit, able to capture all vaccination data because vaccination is delivered free of charge; we also gathered from the hospital's Personal Unit Database the absenteeism data for the periods of July 2017 to June 2018.

As in the previous study, the focus of the present study was on “sporadic absences,” defined as an unplanned sickness absenteeism due to any cause, and as in the previous study, we could not obtain a data set including only ILI‐related and acute respiratory infection (ARI)‐related absences; we still used the same definition for the sake of comparability. This limitation is due to the Italian policy regarding absenteeism records in the workplace, which does not require specifying the medical diagnosis on the absence certificate. The absence is certified by the general practitioner who also establishes the duration.

Once all the data were obtained, they were merged into a single database to work with a comprehensive database. For every employee, a set of attributes was available for further stratifications (eg sex, age, job category, workplace).

The data were divided into the “epidemic period,” starting from week 42 of 2017 to the week 17 of 2018 (28 weeks), and the “non‐epidemic period,” which included by the period from the 26th to 41st week of 2017 and from the 18th to the 26th week of 2018 (24 weeks), and this period was used as the baseline.

Individual sickness absenteeism data were grouped for each of the following job categories: (i) medical doctors; (ii) technical executives (ie pharmacists, dieticians and chemists); (iii) nurses and allied health professionals (ie radiographers, therapists and laboratory technicians); (iv) other executives (ie engineers, lawyers, analysts and statistical and administrative staff); (v) non‐medical support staff (ie ward assistants and cleaning staff); and (vi) administrative staff.

The overall personnel were also grouped into two categories (in‐contact and no‐contact) depending on the nature of their work relationship with patients. The workers were grouped by actual working activity, regardless of the job categories. The “in‐contact” category included all workers who were engaged in direct contact with patients during admission, diagnosis, treatment and/or follow‐up. The “no‐contact” category included all workers who did not work in proximity to patients.

The study protocol was approved by the Directorate‐General of AOU (Prot n. 120 615 del 12/12/2016), and the ethics committee determined that the study did not need their approval.

### 2.2 Statistical analysis

To analyse the data, a custom‐designed computational pipeline was built in the R framework.^21^

Risk analysis was computed using the epiR^22^ package on each strata of all the possible predictors. For each predictor, several contingency tables have been built to compare any remaining strata against a common reference to keep risks within a predictor comparable to each other. Risks are reported with their 95% confidence intervals. Each contingency table so far computed has been tested against Fisher's exact test for count data to better assess the overall robustness of the result. Risk associated to each stratum with different exposition (ie different period and vaccination) has been used to compute the risk difference associated to each stratum for the given exposition. We enforced those results by testing the underling real data distributions with a Student's t‐test, in this case significative p‐values confirm that the differences in absenteeism distribution are statistically significant when tested against different predictors. An overall test at predictor level has been computed (t test for bi‐class predictor, as male/female, and chi‐Square for multi‐class predictors, as age) to further asses the overall reliability of the conclusion.

To check for any possible confounding effect, each predictor has been tested against all the others to identify possible confounders. Three regressive linear models have been built for each predictor, i) the null model with only the predictor as independent variable, ii) a model with the predictor and a linear combination of all the other possible confounders and iii) a third model which also includes mutual effects among all the predictors. To uniformly compare models and avoid intercept biases, we compared the F‐score associated to the predictor across the models and checked for meaningful variations (ie > 10%).^23^, ^24^ All models so far computed do not show any confounding effect with very limited F‐score variations ~ 3%.

All reported p‐values were set with a significance levels at <0.05.

## 3 RESULTS

The number of HCWs at the target hospital was 5,287 during the 2017‐18 study year. Most employees were female (73.7%), mainly nurses and allied health professionals (56.3%), were aged between 40 and 59 years (71.1%) and worked in direct contact with patients (58.4%), and the vaccination coverage was very low among HCWs, only 358 workers (6.8%), as shown in Table 1.

**Table 1:** Description of the study population's characteristics during 2017/18 flu season

Characteristics	Total	Vaccinated	Unvaccinated
Sex	n° (%)	n° (%)	n° (%)
Male	1390 (26.3%)	136 (9.8%)	1254 (90.2%)
Female	3897 (73.7%)	222 (5.7%)	3675 (94.3%)
Age			
<40	546 (10.3%)	20 (3.7%)	526(96.3%)
40‐49	1493(28.2%)	80 (5.4%)	1413 (94.6%)
50‐59	2167 (41%)	145 (6.7%)	2022 (92.3%)
>59	1081 (20.4%)	113 (10.5%)	968(89.5%)
Job category			
Medical doctors	762 (14.4%)	100 (13.1%)	662 (86.9%)
Technical executives	85 (1.6%)	8 (9.4%)	77 (90.6%)
Nurses and allied health professionals	2764 (52.3%)	111 (4.0%)	2653 (96.0%)
Other executives	34 (0.6%)	3 (8.8%)	31 (91.2%)
Non‐medical support staff	1113 (21.1%)	95 (8.5%)	1018 (91.5%)
Administrative staff	529 (10%)	41 (7.8%)	488 (92.2%)
Job in contact with patients			
Yes	3085 (58.4%)	162 (5.2%)	2923 (94.8%)
No	2202 (41.6%)	196 (9.7%)	2006 (90.3%)
Vaccination			
Yes	358 (6.8%)	358 (100%)	‐
No	4929 (93.2%)	‐	4929 (100%)

**Figure 1:**
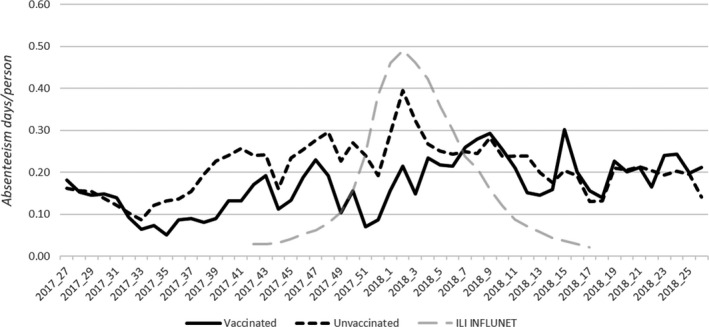
Comparison of the absenteeism rates trend between vaccinated HCWs and unvaccinated during a severe seasonal influenza epidemic 2017/2018 (absenteeism days per person)

The total days lost during the severe intensity influenza season were 56 910, and there was a difference between the epidemic period (35 369 days) and non‐epidemic period (21 541 days). The average number of the days lost for each week was 1094; if we consider the two periods separately, the average days lost per week was 1263 for the epidemic period and 898 days for the non‐epidemic period, with a peak of 2027 days lost in the 2nd week of 2018. Figure 1 shows the rate between the number of days lost per week and the number of HCWs; the two lines represent vaccinated and the unvaccinated personnel. In Italy, vaccination was offered and received by the HCWs between October and November, and consequently, the protection from vaccination was expected at least from the start of the epidemic peak. There was a large difference during the flu peak between these two populations; the rate of the days lost was almost half for vaccinated personnel (0.22 vs 0.40, *P* = .02).

The data gathered from the Ministry of Health showed that during the epidemic period, the peak was reached during the 2nd week of 2018, with an incidence of 14.3/1000 (ILI‐ARI cases/1000 general practitioners’ patients). The data from the regional epidemiologic service (SEREMI) for the Piedmont showed a peak around the 1st week of 2018, with an incidence of 18.9/1000.

Absenteeism among hospital workers during the epidemic period of severe influenza in Italy showed an average increase of +2.63 days/person. Compared with the average of absenteeism during non‐epidemic periods, used as baseline data, this absolute increase correlated with a relative increase of 70% (from 4.05 to 6.68 days/person *P* < .01).

This study shows a significant excess of absenteeism for both female and male, and the excess of female’ absenteeism was almost double that of male absenteeism (2.98 days/person versus 1.70 days/person). Looking at the age classes, the excess of absenteeism showed no large differences among them (Table 2).

**Table 2:** Absenteeism difference among the epidemic and non‐epidemic periods 2017/18 (absenteeism days per person)

Characteristics	Non‐epidemic period		Epidemic period		Excess absenteeism (means difference)	*P*‐value*
	N.	95% CI	N.	95% CI		
Sex						
Male	2.7	(2.22, 3.17)	4.40	(3.79, 5.02)	1.70	*P* < .01
Female	4.52	(4.20, 4.85)	7.50	(7.07, 7.94)	2.98	*P* < .01
Age						
<40	2.57	(2.05, 3.09)	5.15	(4.26, 6.04)	2.58	*P* < .01
40‐49	3.53	(3.1, 3.99)	6.13	(5.47, 6.78)	2.6	*P* < .01
50‐59	4.24	3.81, 4.66)	6.97	(6.39, 7.55)	2.73	*P* < .01
>59	5.11	(4.34, 5.87)	7.68	(6.81, 8.54)	2.57	*P* < .01
Job category						
Medical doctors	1.20	(0.74, 1.77)	1.96	(1.37, 2.55)	0.76	*P* = .07
Technical executives	1.54	(0.42, 2.67)	3.89	(1.28, 6.51)	2.35	*P* = .10
Nurses and allied health professionals	5.02	(4.63, 5.42)	8.19	(6.67, 8.71)	3.16	*P* < .01
Other executives	0.82	(0.27, 1.38)	2.73	(0.18, 5.29)	1.91	*P* = .145
Non‐medical support staff	3.84	(5.23, 4.45)	6.47	(5.63, 7.33)	2.63	*P* < .01
Administrative staff	4.05	(3.28, 4.84)	6.81	(5.76, 7.88)	2.76	*P* < .01
Job in contact with patients						
Yes	4.47	(4.11, 4.83)	7.48	(6.99, 7.94)	3.01	*P* < .01
No	3.45	(3.04, 3.86)	5.58	(5.05, 6.12)	2.13	*P* < .01
Vaccination						
Yes	3.49	(2.56, 4.43)	5.23	(4.16, 6.31)	1.74	*P* = .01
No	4.08	(3.80, 4.37)	6.79	(6.41, 7.17)	2.71	*P* < .01

* Student's *t* *P*‐value refers to the comparison between non‐epidemic and epidemic periods.

^±^ 95% CI, 95% confidence interval.

The average level of absenteeism during the epidemic period increased for all job categories (Table 2), from +0.76 to a maximum of +3.16 days/person, and it was significant for nurses and allied health professionals, non‐medical support staff and administrative staff. Medical doctors were the job category with the least excess absenteeism (0.76 days/person), and all other categories were from 3 to 4 times higher. The peak was among nurses and allied health professionals (3.16 days/person), and they also registered the highest absenteeism during the epidemic period (8.19 days/person). Being in contact with patients was associated with higher excess absenteeism (3.01 days/person, *P* < .01) in comparison with workers who had no contact with patients.

The personnel vaccinated for flu had lower instances of absenteeism in comparison with non‐vaccinated personnel (1.74 vs 2.71 days/person), when compared against their baseline (non‐epidemic period) and the difference was statistically significant.

The results showed a significant and greater excess of absenteeism during severe seasonal influenza compared with excess during moderate seasonal influenza (+0.75 days/person, *P* = .03).

**Figure 2:**
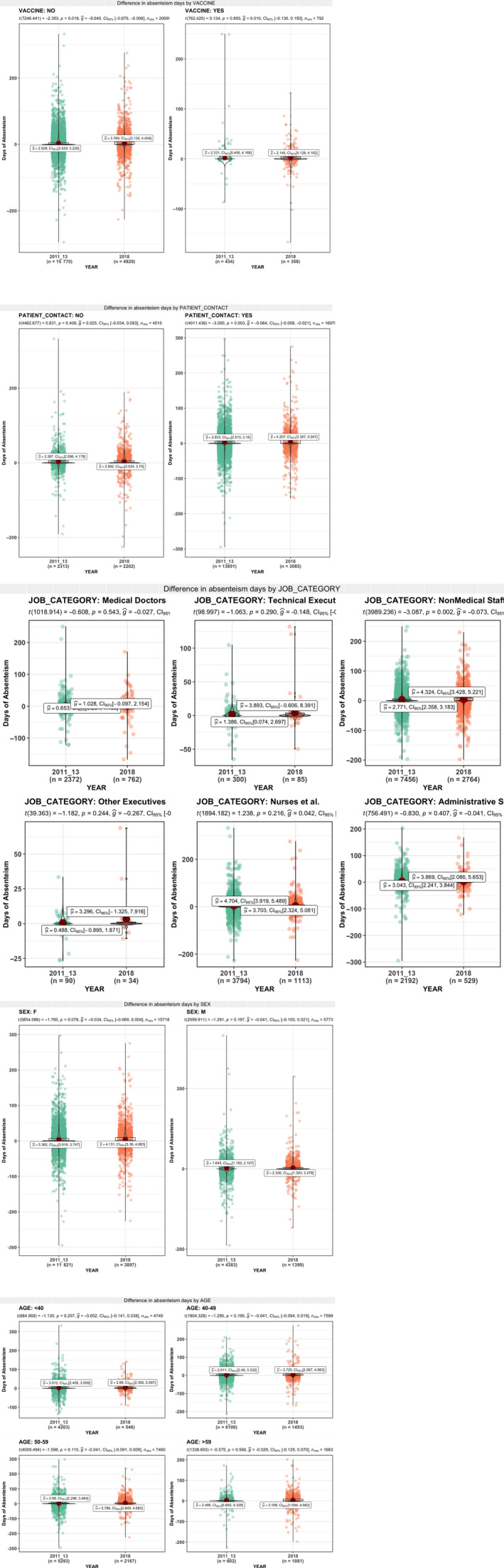
Comparison of excess of absenteeism between moderate and severe seasons (absenteeism days per person)

The absenteeism during moderate and severe seasonal influenza changed for all the characteristics but in a different way. The absenteeism increased during severe seasonal influenza in comparison with moderate seasonal influenza, and there was a significant difference among workers in contact with patients (+1.19, *P* < .01), nurses and allied health professionals (+1.55, *P* < .01) and workers unvaccinated (+0.84, *P* = .01) (Figure 2). The absenteeism also increased for other characteristics but there was not a significant difference (Figure 2) with least increase among medical doctors (+0.38 days/person, *P* = .54) and with except for non‐medical support staff showing a decrease (−1.00 days person, *P* = .216).

## 4 DISCUSSION

The aims of this study were to quantify the increase in absenteeism among HCWs at a large Italian hospital that occurred during severe intensity seasonal flu periods; to estimate the different impacts that moderate intensity flu epidemics and severe intensity epidemics can have on HCWs’ sick absenteeism; and to examine the hypothesis that Italian health workers’ influenza vaccination rates are associated with reduced sickness absence.

The flu season 2017/18 can be considered to be of severe intensity, and it is shown by the data collected from SEREMI and the INFLUNET^18^ that it can be matched in terms of incidence to the pandemic flu of 2009.^18^

Our study showed that there was an increase in absenteeism among hospital workers during this epidemic period of severe influenza in Italy compared with the average of absenteeism during non‐epidemic periods, used as baseline data.

The importance of vaccination of health workers has been proven in different studies.^25^, ^26^, ^27^, ^28^, ^29^, ^30^, ^31^ This study showed a significant difference between the vaccinated group and the unvaccinated group. Although the vaccination rate was very low (6%) in comparison with other studies,^32^, ^33^, ^34^ there was a large difference in the rate of absenteeism during the flu period and in the excess of absenteeism. This result is also highlighted in Figure 1 during the peak of the flu season [week 52nd to 2nd], when the absence rate in vaccinated personnel was almost half that of unvaccinated personnel, even if in the period before the peak (34‐40 weeks) a difference, that cannot be fully explained by the presence or absence of vaccination, was already present. Another important issue to consider is the vaccine used. Indeed, type B (about 60%) and type A (about 40%) flu were circulating in Italy, and mainly B/ Yamagata and A/H1N1 of influenza B and A, respectively. The vaccine used was that containing the four virus strains recommended by the WHO.^35^ The choice of this vaccine avoided the mismatch error that can mislead the analysis and reduce the effect of the protection among the population.^36^ Indeed, based on the circulating type of virus and the type of vaccine, the effectiveness of the vaccine itself is sustainable.

Our findings demonstrated that a severe intensity epidemic can have a higher impact on HCWs’ sick absenteeism in comparison with a moderate intensity flu epidemic and confirm the hypothesis that different intensity epidemics influence the absenteeism of HCWs. This result is consistent with the results of other studies that showed that sick absenteeism is higher during a pandemic compared to seasonal epidemics,^9^, ^37^ and our more modest excess is affected by the fact that we compared severe epidemics (not pandemic) with a moderate seasonal epidemic.

Our results showed that the higher excess of absenteeism among HCWs was not homogeneous among the categories of all of the characteristics of the health workers analysed.

Indeed, the excess of absenteeism among workers in contact with patients showed an increase during severe seasonal influenza in comparison with moderate seasonal influenza period. This overall behaviour is not present in the population of workers not in contact with patients.

This result may be explained by the fact that workers in contact with patients had less compliance with vaccination policies, which in turn results in a higher chance to be exposed to illness.

Our results showed that nurses and allied health professionals category had the highest excess absenteeism in the severe period (+3.16 days/person) and an overall large increase when compared to the moderate seasonal influenza period. This result may be biased by the fact that most employees were nurses, they are the less vaccinated group, and due to their high number they are easier to replace.

This assumption is supported by the literature that reported that people tend to postpone their return to work when they are aware that they can be easily replaced at their job. This especially applies to the Italian health system for nurses, since they are provided with a “backup line” when they are on sick leave.^38^, ^39^, ^40^

Another important result was that the medical doctor category showed the smallest exces**s** absenteeism rate in the severe period and a low and not significant increase in comparison with the moderate period excess absenteeism rate. Several reasons can explain these results. First, vaccination may play a large role in this result because the medical doctor category was the most vaccinated group. Second, it is likely that low absenteeism can be an indicator of presenteeism among the medical staff, which is the phenomenon in which an employee goes to work despite feeling so ill that sick absenteeism would have been appropriate.^41^ Even though sickness presenteeism is common in all working populations, studies have shown that its prevalence increases in the care sector and among healthcare workers likely because the service they provide is customer contact in its nature and compels to the high physical presence requirement.^42^, ^43^ Therefore, the small excess of absenteeism of medical doctors may be because they are educated workers and may be more likely to have high responsibility assignments and to have high job attendance requirements. These suggestions were consistent with the findings of other studies,^44^, ^45^, ^46^ which demonstrated that the level of education of participants and responsibility assignments for relatively more educated participants were significantly associated with sickness presenteeism. Another possible explanation might be due to staff scarcity and high specialization; nobody may be available to cover the work of others apart from the assigned persons for that specific job activity. Indeed, Italian health policy, which blocked the hiring of new doctors and did not guarantee the turnover of those who retired, led to a reduction in the number of working doctors. Furthermore, the Italian health policy guarantees a lower number of specialized doctors than the number necessary to account for those who retire. This suggestion is supported by studies reported from Sweden^44^, ^47^ and another study from Canada^48^ that showed a significant association between workers’ ill presence and lack of staff replacement availability due to understaffing.

An unexpected result is that related to the reduction of absenteeism of the non‐medical support staff job category during severe seasonal flu with respect with moderate seasonal flu. There is a lack of research exploring the association between this job category and sickness presenteeism. It is, therefore, difficult to interpret this finding.

Some limits of our study are due to the definition of absence that we used due to the Italian legislation, as we already stated in the Method sections. It is not possible to verify the illness causing the sporadic absence, which may create some noise during the analysis of the epidemic period; however, there is a clear correlation between the absenteeism peak and epidemic flu. This limitation was inherent in the previous study but guarantees the comparability of the data between the two studies, supported by an unchanging state in sick time policy.

The low rate of vaccination did not allow us to perform a more in‐depth analysis stratifying for vaccination, and we hope that with further work on the coverage levels in the next few years that we will be able to analyse these two groups better. Although the vaccination coverage was very low, only 6.8% of the personnel was vaccinated, we need to assess why this number doubled from that of the previous years. This result was obtained thanks to the effort of the General Direction of the Hospital. Indeed, the vaccination campaign of 2017‐2018 had some innovations, such as the abolishment of a mandatory appointment to get vaccinated, prolonged and dedicated time for flu vaccination in the department of occupational health, and advertisement posters spread in key points of the hospital were displayed to inform all the personnel about the vaccination programme. Although there was a low rate of vaccination, a positive effect of vaccination on absenteeism is present and may support healthcare facilities in taking this choice for their workers.

Finally, to enforce statistical test and better assess population comparability among periods, we tested all the computation on a single‐strata basis. This method guarantees that differences in predictor's strata distribution do just account for small differences in subpopulation sizes, which not affect the overall results, and allow to better handle strata differences caused by small fluctuation and time‐based drift (eg population drift to older age strata).

## AUTHOR CONTRIBUTION

**Maria Michela Gianino:** Conceptualization (lead); Formal analysis (lead); Methodology (lead); Supervision (lead); Writing‐original draft (lead); Writing‐review & editing (lead). **Omar Kakaa:** Conceptualization (supporting); Data curation (lead); Formal analysis (supporting); Writing‐original draft (equal). **Gianfranco Politano:** Data curation (lead); Formal analysis (lead); Methodology (equal); Software (lead). **Antonio Scarmozzino:** Conceptualization (supporting); Data curation (equal). **Alfredo Benso:** Data curation (lead); Formal analysis (lead); Methodology (equal); Software (lead). **Carla Maria Zotti:** Conceptualization (lead); Formal analysis (lead); Methodology (lead); Supervision (lead).

## POTENTIAL CONFLICTS OF INTEREST

All the authors report no conflicts of interest relevant to this article.
